# Dynamics of Photo‐Induced Surface Oxygen Vacancies in Metal‐Oxide Semiconductors Studied Under Ambient Conditions

**DOI:** 10.1002/advs.201901841

**Published:** 2019-09-30

**Authors:** Daniel Glass, Emiliano Cortés, Sultan Ben‐Jaber, Thomas Brick, William J. Peveler, Christopher S. Blackman, Christopher R. Howle, Raul Quesada‐Cabrera, Ivan P. Parkin, Stefan A. Maier

**Affiliations:** ^1^ The Blackett Laboratory Department of Physics Imperial College London London SW7 2AZ UK; ^2^ Department of Chemistry University College London 20 Gordon St London WC1H 0AJ UK; ^3^ Chair in Hybrid Nanosystems Nanoinsitute Munich Faculty of Physics Ludwig Maximilians Universität München 80539 München Germany; ^4^ Department of Forensics Science King Fahad Security Collage Riyadh 11461 Saudi Arabia; ^5^ School of Chemistry Joseph Black Building University of Glasgow Glasgow G12 8QQ UK; ^6^ Defence Science and Technology Laboratory Porton Down Salisbury Wiltshire SP4 0JQ UK

**Keywords:** defects, oxygen vacancy dynamics, surface‐enhanced Raman spectroscopy (SERS), titanium oxide

## Abstract

Surface‐enhanced Raman spectroscopy (SERS) is a powerful analytical technique commonly used in the detection of traces of organic molecules. The mechanism of SERS is of a dual nature, with Raman scattering enhancements due to a combination of electromagnetic (EM) and chemical contributions. In conventional SERS, the EM component is largely responsible for the enhancement, with the chemical contribution playing a less significant role. An alternative technique, called photo‐induced enhanced Raman spectroscopy (PIERS) has been recently developed, using a photo‐activated semiconductor substrate to give additional chemical enhancement of Raman bands over traditional SERS. This enhancement is assigned to surface oxygen vacancies (*V*_o_) formed upon pre‐irradiation of the substrate. In this work, the exceptional chemical contribution in PIERS allows for the evaluation of atomic *V*_o_ dynamics in metal oxide surfaces. This technique is applied to study the formation and healing rates of surface‐active *V*_o_ in archetypical metal‐oxide semiconductors, namely, TiO_2_, WO_3_, and ZnO. Contrary to conventional analytical tools, PIERS provides intuitive and valuable information about surface stability of atomic defects at ambient pressure and under operando conditions, which has important implications in a wide range of applications including catalysis and energy storage materials.

## Graphical abstract

Oxygen vacancy defects play significant roles in altering the overall reactivity and functionality of metal oxides affecting both bulk and surface properties, even at very low concentrations. Surface vacancies have only previously been studied under far from operando conditions. Through photo‐induced enhanced Raman spectroscopy, a method to indirectly track the dynamics of photo‐induced surface oxygen vacancies is presented.

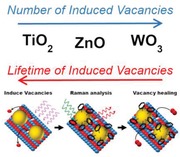

Metal oxides are extensively used in a wide range of industrial applications such as catalysis, electronics, and energy storage.^1^, ^2^, ^3^, ^4^ These materials can contain several types of defects, such as metal interstitials, metal vacancies, and oxygen vacancies (*V*_o_), resulting from their synthesis route and post treatment. Such defects can drastically alter the chemical and physical properties of functional materials, playing an important role in their overall performance.^3^, ^5^, ^6^, ^7^, ^8^, ^9^, ^10^ For instance, *V*_o_ can become some of the most reactive sites on the material surface^11^ and greatly influence the materials' photocatalytic activity.^12^, ^13^ Thus, it is not surprising to find *V*_o_ have been studied extensively, with recent computational studies dedicated toward understanding oxygen vacancy dynamics, their energy of formation,^14^, ^15^ and electronic structure.^6^, ^16^ Unfortunately, due to the inherent low concentrations and high reactivity of *V*_o_, experimentally probing vacancy sites in materials is challenging, particularly under ambient conditions. As a result, to the best of our knowledge, experimental production and healing rates for *V*_o_ have not been directly observed. Vacancy states have been widely studied using electron paramagnetic resonance (EPR) and X‐ray photoelectron spectroscopy (XPS),^17^ however these techniques typically operate at ultra‐high vacuum conditions and/or cryogenic temperatures, i.e., far from operando conditions for most applications. On the other hand, scanning probe techniques such as atomic force microscopy (AFM),^18^ scanning tunneling microscopy (STM),^19^ and Kelvin probe force microscopy^20^ have recently been used to investigate the migration of vacancies, *V*_o_ healing, and controlling vacancy concentrations at ambient conditions. These techniques often require very flat substrates, typically single crystals, and operate at the atomic scale, making it challenging to extrapolate any observation to microstructured materials for practical applications. Thus, there is great demand for new methods of measuring the thermodynamics and the kinetics of *V*_o_ production and loss on realistic, textured substrates and under ambient or other operando conditions, at the macro scale.

Surface‐enhanced Raman spectroscopy (SERS) is a powerful and sensitive analytical technique widely applied across chemical and biochemical sensing, including in the field of catalysis.^21^ In SERS, the Raman scattering of molecules adsorbed on a rough, nanostructured, plasmonic substrate may be enhanced by several orders of magnitude, enabling the detection of single molecules.^22^, ^23^, ^24^ This enhancement is attributed mainly to the intense electromagnetic (EM) field confinement provided by the excitation of localized surface plasmonic resonance (LSPR). It has also been shown that chemically absorbed molecules on nonplasmonic substrates can be enhanced due to changes in charge distribution and vibronic states, termed chemical enhancement (CE).^25^, ^26^, ^27^ In conventional SERS, the CE phenomenon is usually weaker than the EM contribution. However, recent reports have shown comparable CE and EM enhancements using semiconductor materials. Cong et al.^28^ reported recently on a defective oxide, showing significant enhancements, and through oxygen incorporation Zheng et al. demonstrated similar enhancements.^29^ Our group demonstrated that CE could be induced using UV‐irradiation of TiO_2_ rutile substrates and then coupled with EM‐SERS by depositing Au or Ag nanoparticles (NPs) to generate a useful enhanced signal. The technique was termed as photo‐induced enhanced Raman spectroscopy (PIERS).^30^ It is hypothesized that the changes to the local environment (charge and reactivity) directly impact on the charge distribution and vibronic states of the Raman probe, causing the observed differences in CE enhancement. Adopting this idea, sub‐zeptomole concentrations of analytes could also be detected through similar combination of EM with CE enhancements on organic, rather than inorganic semiconductors.^31^

In the PIERS sensing scheme, high‐energy photons (UVC) are used to expel oxygen atoms from the surface of the material (possibly mediated by a photoreaction with adsorbed H_2_O and O_2_),^19^ increasing *V*_o_ concentration. The generation of these atomic‐scale defects enables enhanced Raman transitions due to resonant conditions—also known as photo‐induced charge‐transfer processes—between the defect semiconductor substrate and the probe molecules adsorbed on the AuNPs. Some authors have also described this process as vibronic coupling of resonances in the metal–molecule–semiconductor system.^10^, ^27^ Photo‐inducing *V*_o_ results in a very small concentration of surface *V*_o_, in comparison to other vacancy incorporation methods. Although Raman bands are enhanced with the presence of vacancies alone, the small *V*_o_ concentration in combination with the CE mechanism generally having a weaker contribution to SERS often results in it being very difficult to distinguish such changes without the presence of AuNPs. Aside from providing an additional EM enhancement mechanism, the metal–semiconductor interface is thought to also help to stabilize the formation of oxygen vacancies, reduce the vacancy formation energy, and increase charge carrier separation lifetimes.^32^ As such, this scheme provides synergistic enhancement over the Raman scattering signal, accounting for both EM (AuNPs) and CE (*V*_o_) enhancements.^30^ Here, we demonstrate that the induced CE contribution to the total Raman intensity signal can serve as a local probe to track the dynamics of atomic defects in TiO_2_, WO_3_, and ZnO. Thermodynamic and kinetic information of *V*_o_ generated in situ could be extrapolated from UV exposure time and relaxation time at ambient conditions upon correlation of Raman enhancements. Photo‐induced surface vacancy concentrations were found to be too small to be measured with some alternative techniques, demonstrating the sensitivity of PIERS. Dynamic tracking of these atomic defects at ambient conditions could impact a wide range of areas such as material science and energy storage, among others.

In a slightly modified scheme, based on PIERS work by our groups and others,^30^, ^33^, ^34^ we collected Raman spectra of pre‐functionalized AuNPs coated with mercaptobenzoic acid (MBA), henceforth AuNPs‐MBA, drop‐cast on top of different metal oxide thin films, namely, TiO_2_ (rutile), ZnO (wurtzite), and WO_3_ (monoclinic). Although MBA has an excellent Raman cross‐section, good water solubility, and in addition to being a polar aromatic thiol, the PIERS mechanism is nonspecific to the choice of analyte. Due to these qualities, MBA has often been used as an attractive candidate as a universal probe for modeling different surfaces, hence the choice as the reporter molecule in this study. Pre‐functionalization of the AuNPs creates an effective self‐assembled monolayer of MBA on the AuNP surface (bound by a thiol‐Au bond as shown in **Figure** **1**). However, due to the highly focused EM fields produced by LSPR, only molecules within the hot‐regions on the particle, or between particles, are likely dominate the resultant Raman spectra. At the metal–semiconductor interface, additional enhancement can occur due to preferential concentration of local electric field. Although each of these factors will contribute to the SERS signal, the greatest changes in band intensities over time, primarily through the PIERS enhancement and decay, will occur for MBA molecules at the metal–semiconductor interface in close proximity to induced vacancy states, as shown in the schematic in Figure 1. By normalizing the measured Raman spectra with respect to the average SERS baseline, all variables are effectively constant except for effects arising from *V*_o_ states. Raman spectra were taken before, during and after UV treatment of the substrates, however it is important to note the UV lamp was switched off while the Raman spectra were acquired due to experimental constraints. Hence, the measured Raman spectra would not exhibit any enhancement affects resulting from changes in the interfacial properties caused by the UV light whilst measurements were taken. Pre‐functionalization of the Raman probes on AuNPs also prevented the probe molecule reacting with the highly active photocatalytic surface. As such we observed no degradation over time from UV exposure. This is contrary to the previously reported work,^30^ where molecules were directly drop‐cast over PIERS substrates and UV exposure removed the probe molecules. In all cases, the band intensities were enhanced above the SERS spectral intensity during and immediately after UV irradiation, which has been attributed to the formation of *V*_o_ in the PIERS mechanism.^30^ Figure 1b shows the typical MBA bands at 1065 and 1575 cm^−1^ which are attributed to *v8a* and *v12* aromatic ring vibrations, respectively.^35^ The characteristic bands of TiO_2_ (at 438 and 602 cm^−1^) and WO_3_ (at 768 and 939 cm^−1^) (Figure S1, Supporting Information) were observed in corresponding studies and used as reference bands for normalization, since they did not change over time and prolonged irradiation (Figure S2, Supporting Information).

**Figure 1:**
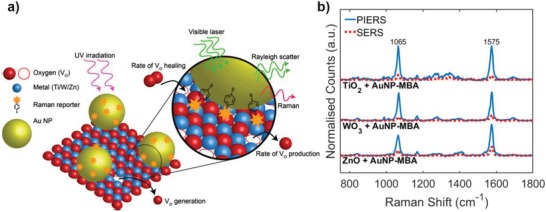
a) Schematic of the PIERS technique to probe vacancy dynamics: irradiation with UV light creates photo‐induced vacancy states which interact with AuNPs and the Raman reporter (mercaptobenzoic acid (MBA) as a surface monolayer) enhancing scattering for Raman detection. b) Raman spectra showing the strongest AuNP‐MBA bands at 1065 and 1575 cm^−1^ on different metal‐oxide films before (red dashed line, SERS) and after (blue solid line, PIERS) UV irradiation. Note the increased intensity of the MBA Raman peaks after oxygen vacancy generation.

After UV irradiation, the PIERS signal decayed back to the baseline SERS signal as a result of healing of the surface upon exposure to air (relaxation time). A control sample of TiO_2_ was pre‐irradiated under the same conditions and immediately stored under vacuum for 2 h, before deposition of AuNP‐MBA and Raman analysis. Figure S2 in the Supporting Information shows that the average Raman intensity on removal from the vacuum is comparable to that of PIERS, subsequently decreasing in intensity upon exposure to air. This is consistent with our explanation of the PIERS mechanism driven by photo‐induced *V*_o_, since these defects would not be able to heal under vacuum conditions. Although both metal and oxygen vacancies can be induced, these results suggest *V*_o_ are the predominant type of induced defect in both the TiO_2_ and WO_3_ substrates. Due to the nature of ZnO, it is less evident that oxygen vacancies should be the dominantly produced species. However, recent studies have shown, while the zinc vacancy can commonly occur, the *V*_o_ in ZnO has a lower formation energy^36^ and is also the predominant ionic defect.^37^ Hence, we infer the dominant enhancements in the Raman spectra shown are due to induced *V*_o_ for all investigated cases.

A series of Raman spectra were collected in situ every 3 min upon UV irradiation (*V*_o_ generation) at the same position on each of the substrates (**Figure** **2**a–c). In these measurements, an initial decrease in band intensities, which was attributed to laser‐induced degradation of MBA molecules, was widely observed for the three substrates. Controls showed negligible changes in band intensity after a long UV exposure period, hence band intensity decreases due to photolysis were assumed to be negligible. During this activation period, typically 5 min of continuous UV irradiation, *V*_o_ formation was assumed to be negligible so as not to cause a noticeable PIERS enhancement. Other effects involving adsorbed water/oxygen molecules, such as water splitting, may take place during the activation period. After this period, significant band enhancements were detected for the three substrates, however their corresponding rate profiles were very different within the irradiation time considered (30 min). For TiO_2_ substrates, band enhancement was found to increase steadily upon continuous irradiation, reaching a maximum enhancement value after 20–25 min (Figure 2g), indicating an equilibrium between enhancing and decay mechanism, i.e., *V*_o_ formation and healing, respectively. WO_3_ substrates were found to have a steady increase in intensity initially. After around 20 min a more rapid increase in intensity was found to occur for WO_3_ substrates than observed in the TiO_2_ and ZnO films (Figure 2h).

**Figure 2:**
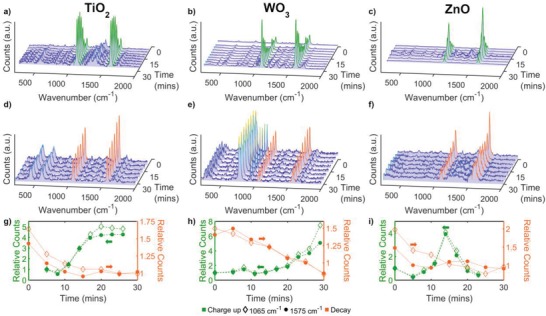
Raman spectra of AuNPs‐MBA on TiO_2_, WO_3_, and ZnO (first to third columns, respectively) upon a–c) continuous UV exposure over 25 min and d–f) after healing in the dark until the enhancement returned to the SERS background signal, around 30 min. g–i) Corresponding changes in relative band intensities, 1065 cm^−1^ (empty symbols) and 1575 cm^−1^ (full symbols) over time are plotted in the lower row. Green and orange colors correspond to data upon UV irradiation (*V*_o_ formation, *V*_o_^+^) and decay upon exposure to air (*V*_o_ healing, *V*_o_^−^), respectively.

The ZnO films did not show consistent behavior across samples. Despite enhancement of the Raman bands at some measured positions (while under UV exposure), at other positions, on the same sample, sharp spikes in intensity subsequently followed by rapid decay in enhancement were also found (Figure 2i). However, often an overall increasing trend was generally observed, even with the fluctuations in band intensity (Figure S3, Supporting Information). The rapid enhancements found could be explained by the microstructure and crystallinity of the ZnO films used. The ZnO films, in comparison to the other films studied, were generally much less crystalline (Figure S8, Supporting Information) and appeared to have a much smoother surface structure (Figure S9, Supporting Information), which has been reported to reduce photocatalytic activity and therefore in turn vacancy production. ZnO has a lower Zn‐O bond energy, relative to the other metal oxides,^38^, ^39^ resulting in the likelihood of more vacancy states forming, which can explain the tendency for sudden increases in enhancement. However, the *V*_o_ in ZnO has been predicted to have a charge of 2+, where this positively charged state has been suggested to be unstable on the ZnO surface under thermodynamically stable conditions, hence the rapid decay.^36^ The combination of the microstructure, crystallinity, and Zn‐O bond energy therefore may explain the fluctuating results found in Figure S3 in the Supporting Information. An average of Raman series at many positions across each metal oxide surface was taken to ensure a representative behavior of the film. The importance of signal averaging across both the surface and multiple substrates was further highlighted by the decay in MBA band intensity during UV exposure at some measured points. This unexpected band decay was probably due to the healing of vacancy states surrounding the measured position, without new vacancy states forming, hence a loss of local enhancement.

After the long irradiation period (105 min), an additional series of Raman spectra was collected every 5 min to observe the signal decay upon exposure to air. It was noted that the decay in band intensities, attributed to *V*_o_ healing, occurred at different rates for the three metal‐oxide substrates (Figure 2d–f), as expected given their differing *V*_o_ formation profiles. It should be noted that in addition to the time taken between each measurement, experimental constraints introduced a small time gap (roughly 5 min) between removal from UV exposure and the first recorded Raman measurement. Due to the relatively short lifetimes of the PIERS effect and exponential‐like behavior of the decay, significant enhancement decreases can occur prior to the first few minutes, resulting in the first recorded Raman spectra having a lower enhancement factor that the last recorded “charge‐up” measurement. This is evidenced in Figure 2g–i, where a greater change can be seen under UV exposure in comparison to after UV exposure. For comparison with the Raman series under constant UV irradiation, each PIERS decay series was displayed relative to the time of the first recorded Raman spectra, as opposed to time removed from UV exposure. However, the true times, from removal of UV exposure, were used in the calculations below (and **Figure** **3**).

**Figure 3:**
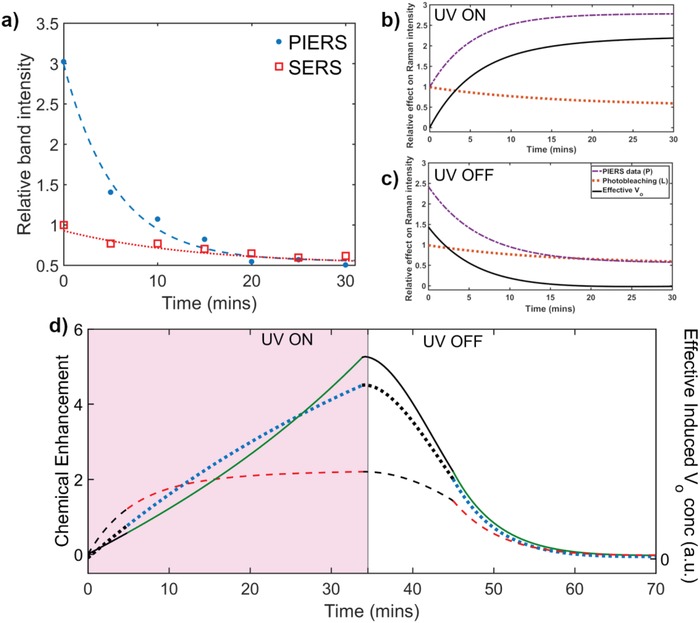
a) Decay of 1065 cm^−1^ MBA band on ZnO with (blue filled dots) and without (red empty squares) UV pre‐irradiation, corresponding to SERS and PIERS effects, respectively. Contributions to the changes in measured Raman band intensities, *P* (purple dashed line), where *P* can be deconvoluted using Equation (1) into the effects from; photo‐bleaching (*L*, orange dotted line), and the contribution due to the effective number of vacancy states, Eff*V*_o_ (black solid line), b) under UV and c) after UV exposure, respectively. d) Chemical enhancement of Raman signal, due to induced *V*_o_, correlated to the effective *V*_o_ concentration over time during UV irradiation (*V*_o_^+^ and *V*_o_^−^ occurring) and subsequent healing (only *V*_o_^−^ occurring) relative to the SERS baseline for TiO_2_ (red dashed line), WO_3_ (green solid line), and ZnO (blue dotted line). Black lines are extrapolations from experimental points.

Time‐dependent changes in the measured PIERS enhancement (*P*) primarily depend on three factors: *V*_o_ formation (*V*_o_^+^) and healing (*V*_o_^−^) dynamics, as well as laser‐induced bleaching (*L*) of MBA molecules. Changes in vacancy concentration are substrate dependent, closely connected to the photocatalytic activity of the substrate hence, the two factors, *V*_o_^+^ and *V*_o_^−^, are assumed to be independent of the probe molecule. In contrast, bleaching of the probe molecule is dominated by light‐matter interactions, amplified by the enhanced electric fields due to the plasmonic NPs, and so was assumed to be independent of the substrate. Although this was presumed, the rate of photo‐bleaching was still calculated independently for each respective metal oxide by exposing the probe on non‐UV‐irradiated samples to the Raman laser in a replicate experimental measurement, and measuring loss in intensity with increasing Raman laser exposure. It is interesting to note that (within experimental error) *L* was found to be the same across all substrates, supporting the proposal that the substrate plays little‐to‐no role here. Under ambient conditions and UV irradiation, induced surface vacancy states can subsequently heal after their formation. Therefore, at any given time the measured Raman bands enhancement results from an effective vacancy concentration (Eff*V*_o_) equal to the difference between the number of vacancy states formed and the number of those states which have been healed. The deconvolution analysis of Raman bands considered with changes attributed to PIERS enhancement (*P*) as described in Equation (1)

(1) $$P=\mathrm{Eff}V_{o}\;+L$$

It should be noted that each variable in Equation (1) is defined as the time‐dependent changes in Raman intensity due to each respective factor. Although each variable presented is thought to correspond to an independent mechanism, and may affect either the semiconductor substrate or analyte molecule separately, ultimately all have a measurable effect on the Raman intensity. An understanding of the physical processes responsible for each respective factor is required for an accurate and meaningful fit to the experimental data, corresponding to physically meaningful values. It is important to note under no UV irradiation Equation (1) simplifies to the standard decay of an SERS signal over time due to laser‐induced photo‐bleaching.

The rate of photo‐bleaching of analyte molecules under laser, during Raman measurements, has been shown to strongly depend on the distribution of Raman cross‐section enhancements.^40^ This was empirically shown to be well described by a simple exponential function. Hence, as the measured changes in Raman intensity over time correspond to changes in Raman enhancement, intensity decreases due to photo‐bleaching, *L*, were fitted to a simple exponential decay. A function of the form *A*e*^−BT^* was chosen, where *A* and *B* are fitting parameters, respectively. In a classic exponential decay, parameter *B* often refers to a rate, related to the decay time constant. In our case, as we are concerned with determining the dynamics of vacancy formation and healing only parameter *B* was used to determine physically meaningful data.

As mentioned above, the Raman band enhancements at any given time will be affected by the effective number of induced *V*_o_ on the surface, Eff*V*_o_. The rate of change of the concentration of induced *V*_o_ states depends directly on the rate at which *V*_o_ are formed (*V*_o_^+^) and the rate at which those *V*_o_ subsequently heal (*V*_o_^−^), Equation (2)

(2) $$\frac{\mathrm{dEff}V_{o}}{dt}=\frac{dV_{o}{}^{+}}{dt}\;+\frac{dV_{o}{}^{-}}{dt}$$

where d*V*_o_^+^/d*t* and d*V*_o_^−^/d*t* represent the rate of vacancy formation and vacancy healing, respectively. It is likely that the mechanism behind vacancy formation and healing is different; hence, we assume these two rates are independent of one another.

As surface vacancy defects are some of the most reactive sites, vacancies often are found to heal after interacting with adsorbed molecules. Although many of these reactions and processes have been widely reported in the literature, experimentally determined dynamics of vacancy healing, to the best of our knowledge, have not been reported. The two most common molecules responsible for vacancy healing under ambient conditions are O_2_ and H_2_O.^41^, ^42^ The concentration of oxygen vacancies has been shown to often relate to the concentrations of water and oxygen, respectively, as a pseudo‐first‐order rate equation.^43^, ^44^ Where no UV irradiation is present, no additional *V*_o_ are induced. In the case where substrates were pre‐irradiated with UV light and subsequently measured with no further UV exposure d*V*_o_^+^/d*t* = 0, and therefore dEff*V*_o_/d*t* = d*V*_o_^−^/d*t* and can be substituted directly into Equation (1). Hence, after *V*_o_^−^ was calculated using Equation (1), it was fit to the integrated rate law for first‐order reactions. This was generally found to empirically fit the data very well.

Although the mechanism for vacancy healing is generally well reported on in the literature, vacancy formation has received much less attention. However, the dynamics of vacancy formation largely depends on the physical process used to induce vacancy states, e.g., thermal‐vacuum annealing, sputtering, and UV treatment. A similar reaction equation to vacancy healing is often reported for vacancy formation.^5^, ^43^ Yet, vacancy formation under UV exposure is a more complicated process, and therefore requires additional consideration. Generally, the UV irradiance upon the metal‐oxide semiconductors results in the formation of charge carriers. These charge carriers interact with adsorbed surface species, such as H_2_O and O_2_, often splitting the molecules creating radical species. The resultant radicals subsequently attack surface bridging oxygen atoms, removing them, and leaving behind a vacancy state. This process has been shown to be enhanced surrounding a metal–semiconductor interface, often resulting in increased vacancy formation.^32^ The rate‐limiting step within the multistep process has been reported to be the initial carrier production, specifically holes.^45^ As a result, *V*_o_^+^ was assumed to be pseudo‐first order as well. This arguably simplified model can explain our experimental observations. However, it is important to note that due to the many factors associated with vacancy production, our model is a good empirical first step, but many other factors could contribute to underlying variance observed.

As an example of this analysis, Figure 3a shows intensities of the MBA band at 1065 cm^−1^ before (SERS) and after (PIERS) UV irradiation on a ZnO substrate. The baseline decay in the SERS signal can be attributed to reporter photo‐bleaching by the Raman excitation laser (*L*) and an average *L* was calculated for each substrate from SERS measurements over 5–10 positions. Measurements of the PIERS decay were conducted after a long UV exposure period (105 min). An equilibrium state of maximum induced vacancies was assumed and after removal of the substrate from the UV source it was assumed no further vacancies were induced during measurements, setting d*V*_o_^+^/d*t* = 0. The PIERS decay was thus attributed to *V*_o_^−^ + *L* and thus *V*_o_^−^ could be obtained from subtraction of *L* from the SERS measurements, Figure 3c (as dEff*V*_o_/d*t* = d*V*_o_^−^/d*t*). Using the measured values for *L* and calculated d*V*_o_^−^/d*t* and the measured band intensities (*P*), it was possible to then estimate d*V*_o_^+^/d*t* on each substrate under UV exposure.

The corresponding set of data obtained following this procedure for the case of a TiO_2_ substrate is shown in Figure 3b,c. The plots presented in Figure 3b,c arise from fitting the experimental data over time for PIERS enhancements and photo‐bleaching (SERS decay) and the calculated effect on the Raman band intensity from Eff*V*_o_ (determined using Equation (1)). The rate of change of enhancement due to vacancy formation and healing was then determined for each respective substrate. Figure S4 in the Supporting Information shows the corresponding rates for the sample analysis shown below in Figure 3b. The calculated Eff*V*_o_, for both UV on and off cases, were then combined to show the complete charging to discharging cycle (Figure 3d), corresponding to the changes in CE. Data were extrapolated between the charge‐up and decay data sets and during the initial 5 min irradiation period, where no measurements could be taken due to experimental constraints (i.e., deposition of AuNPs‐MBA). The left side of Figure 3d shows the increase in CE from the SERS baseline, before UV exposure with no induced vacancy states (*T* = 0 min), and the right side of Figure 3d shows the decay of enhancement after UV exposure returning to the original SERS signal (i.e., when CE = 0 then measured PIERS = SERS). By applying Equation (1) and Equation (2) to the experimental and calculated data for each respective metal oxide, d*V*_o_^+^/d*t* and *d*V_o_^−^/d*t* were then determined (Figure S5, Supporting Information), recorded in **Table** **1**.

**Table 1:** Calculated chemical enhancement factors (EF) and *V*_o_ formation and healing rates for photo‐induced *V*_o_ from measured data for each investigated metal oxide calculated from the related rate of change of Raman band enhancement over time. EF were calculated relative to the SERS intensity

Metal oxide	Average relative PIERS EF	Highest relative PIERS EF	Average *V*_o_ formation rate [min^−1^]	Average *V*_o_ healing rate [min^−1^]	Literature *V*_o_ formation energy [eV]
TiO_2_	3.07	7.39	0.204 ± 0.085	0.183 ± 0.013	4.2^15^
WO_3_	5.11	10.00	0.257 ± 0.045	0.195 ± 0.023	1.45^50^/3.46^47^
ZnO	3.16	6.87	0.254 ± 0.108	0.196 ± 0.021	3.23^51^/3.31^52^/3.5^53^

Series of Raman spectra were also taken under the same conditions for alternative probe molecules on TiO_2_ substrates which showed comparative results for induced vacancy healing rates (Figure S6, Supporting Information). In addition, while the MBA presented here were chemisorbed onto AuNPs, samples where molecules were physisorbed were also tested. The dynamics were also found to be independent of this. By presenting widely different molecules, with significant difference in molecular electronic structure, each molecule‐metal‐semiconductor‐vacancy system has its own unique charge‐transfer possibilities. However, the primary, if not only, time‐dependent change to charge‐transfer effects arise from changes in the surface *V*_o_ concentration. Hence, the comparative PIERS decays for alternative probe molecules (Figure S6, Supporting Information) over time suggest that the mechanism for photo‐induced *V*_o_ production and subsequent *V*_o_ healing does not involve the probe molecule.

Table 1 shows the average and highest relative PIERS enhancements found over all the Raman studies conducted, relative to the SERS baseline. This is equivalent to a measure of the CE as a result of induced *V*_o_. The highest CE values were found for WO_3_ films as a result of most induced surface *V*_o_ (Table 1). WO_3_ films were found to show a slower increase in vacancy concentration over a longer time period under UV exposure, in comparison to the other metal oxides measured. The rate of CE increase appeared to increase steadily past 20 min for WO_3_, where ZnO and TiO_2_ films both showed gradual increase toward a maximum CE. The second highest PIERS enhancement was found with TiO_2_ films, however on average TiO_2_ showed a much lower CE. A direct correlation can be seen between the rate of vacancy formation and average PIERS enhancement found, where CE and d*V*_o_^+^/d*t* of WO_3_ > ZnO > TiO_2_, Table 1. However, at shorter times (<25 min), the average CE in ZnO was found to be higher than WO_3_. Consequently, the average *V*_o_ formation rate for ZnO was found to be on a similar order of magnitude to WO_3_. A steady increase in CE was seen for ZnO appearing to head toward a maximum after 35 min of UV exposure. For longer exposure periods, a greater difference between WO_3_ and ZnO films may be measured. Although TiO_2_ films were found to show a significant initial increase over small times (<10 min), a maximum was reached after 12–15 min. This initial increase was found to be greater than the enhancements of both WO_3_ and ZnO at similar times, yet the average *V*_o_ formation rate reported is lower due to the plateau at 12–15 min. A small increase in the CE can be seen for TiO_2_ under UV exposure over time past 15 min. However, a near equilibrium state appears to have been reached between *V*_o_ formation and *V*_o_ healing, impeding further CE. Other studies showing comparative timescales for UV‐induced changes in measured effects in TiO_2_ films, assigned to oxygen vacancies, were found to be consistent with our results.^33^, ^46^ A comparative trend to *d*V_o_^+^/d*t* can also be seen for *d*V_o_^−^/d*t* (Table 1), where WO_3_ and ZnO films were found to show more comparative average values than that of TiO_2_.

The significant differences in oxygen vacancy healing times for each material suggests that the surface vacancy formation energies for ZnO and WO_3_ are lower than TiO_2_, which is consistent with computational studies^15^, ^47^, ^48^ and reported bond formation energies.^38^, ^39^ The characteristics of vacancy healing appear to follow a similar profile across each metal oxide measured. An induced vacancy can easily be healed under ambient conditions via interacting with surface adsorbed species, such as O_2_ and H_2_O. Additional factors such as metal valency, charge on the induced defect, and microstructuring of surface may also have an effect on the movement and interaction of the adsorbed species with the *V*_o_, affecting the measured healing rate.

The presented Eff*V*_o_ in Figure 3d represent an average behavior over multiple positions on multiple samples. Generally, a similar decay in Raman signal was seen for each metal oxides after initial UV pre‐irradiation. The behaviors of TiO_2_ and WO_3_ under UV appeared consistent across the different samples and positions measured. However, ZnO samples showed varied enhancements upon UV exposure. Some positions on samples of ZnO were found to produce lower CE values than TiO_2_ films under UV exposure, as shown by the maximum CE values (Table 1), with fluctuations in band intensities over short durations (Figure 2i). This result had an impact on the corresponding average enhancement, resulting in what appears to be a more consistent and higher average CE between 10 and 25 min. It is thought that these fluctuations were the result of many vacancy states induced in ZnO due to the low Zn‐O dissociation energy,^39^ resulting in a high CE. Yet despite this, many of the vacancy states induced were believed to be unstable, resulting in a rapid drop in CE, as seen in Figure 2i, as discussed above. However, over a longer exposure period a larger number of more stable *V*_o_ sites were able to form, resulting in the more uniform *V*_o_^−^ decay seen.

Although the reported values are not the absolute rate of change in *V*_o_ concentration, but rather an indirect measure of the effective changes in induced vacancies, the results still present an insight into atomic defect changes on the metal oxide surfaces. It is important to note that a range of energies has been reported in the literature for the three metal oxides measured due to the large number of possible crystal structures positions of the vacancies. Often the production of vacancy states is modeled under different conditions to the experimental measurements. Although a direct comparison between the calculated values and experimental data is generally not possible, the trends in enhancements are strongly supported by formation energies across the metal oxides in the literature.

We have demonstrated that surface oxygen vacancy states can be induced on different photocatalytic metal oxide substrates by photo‐irradiation and monitored by tracking enhanced Raman spectra through the PIERS technique. This allowed an estimation of the vacancy formation and healing lifetimes. Through the use of Raman spectroscopy, in comparison to other techniques for probing *V*_o_ defects, it was possible to monitor changes to the surface in real time conditions. Inducing vacancy states using UV illumination has often been found to induce a very small concentration of vacancies, in comparison to other vacancy induction methods. Although many techniques have been found to detect *V*_o_,^9^, ^49^ often the measurable concentrations caused by UV photo‐irradiation are unable to be detected. Hence, PIERS under these conditions has been shown to be a more sensitive technique for reaction conditions. An additional notable advantage to our measurements, as opposed to other common techniques used to analyze *V*_o_ defects such as XPS and EPR, is the ability to measure under operando conditions. Scanning probe techniques, such as AFM and STM, have been shown to track changes of individual vacancy states, however due to the nature of scanning probe measurements the techniques often require many hours under high vacuum conditions to delivery this sensitivity, where only a nanoscopic section can be scanned. By using PIERS as possible alternative, macroscopic areas can be analyzed in real time within seconds or minutes with the additional advantage of more chemical information available from the Raman spectra. A significant advantage of PIERS over other possible techniques.

WO_3_ was found to produce the most vacancies through this method but was also found to support the shortest average vacancy lifetimes. TiO_2_ was found to have the lowest number of induced *V*_o_ yet the longest lifetime appeared to reach a maximum and stays in an equilibrium state after 15 min of UV exposure. The technique we have demonstrated here may be applied as a more general method to quantify the dynamics of vacancy states through Raman enhancement. Very few studies to date have shown the tracking of the generation and healing of single atomic *V*_o_ over time, primarily used scanning probe methods on flat single crystals. To the best of our knowledge, this is the first approach to track *V*_o_ dynamics on a larger scale, particularly in materials with surface activity for catalysis, making PIERS a potentially valuable technique for understanding vacancy states in catalysts and other functional metal oxide materials. Importantly, PIERS works in a nondestructive manner and has the potential to work on shaped and structured materials, introducing the possibility of operando characterization. Through a greater understanding of the vibronic coupling resonances exploited in PIERS substrates, theory may be used to predict enhancement factors and sensitivity for industrial applications of the PIERS process. After advances within the SERS and plasmonics fields in standardization of methods and processes measured by Raman spectroscopy, it is possible that PIERS enhancements could be used as a direct method of surface *V*_o_ concentration quantification. Further investigation is ongoing, employing different methods of producing catalytic vacancies and defects to extend this technique beyond photocatalysis.

## Experimental Section

*Synthesis of TiO_2_ Thin Films*: TiO_2_ thin films were synthesized using aerosol‐assisted chemical vapor deposition (AA‐CVD). Titanium (IV) isopropoxide (Sigma‐Aldrich, 97%, 0.5 mL) and methanol (Fisher Scientific, HPLC grade, 20 mL) were used as the precursor and solvent, respectively. The precursor solution was placed under nitrogen (BOC) flow, to be used as the carrier gas, where the gas flow rate was set to 1 L min^−1^. An ultrasonic humidifier (Liquifog, Johnson Matthey, operating at 2 MHz) formed an aerosol of the solution and the precursor was transported using nitrogen carrier gas. The typical growth rate of TiO_2_ under these conditions was ≈10 nm min^−1^.^54^ The CVD reactor, which was consisted of a 320 mm long graphite heating block, was accommodated in a quartz tube with three Whatman heater cartridges. The temperature of the entire system was controlled by Pt‐Rh thermocouples. The TiO_2_ films were deposited at 500 °C on quartz slides (15 × 15 × 1 mm), purchased from GPE scientific, and then annealed to 1000 °C for 10 h to obtain the rutile phase. The presence of pure rutile was confirmed by X‐ray diffraction and Raman spectroscopy and no traces of anatase were detected.

*Synthesis of ZnO Thin Films*: ZnO thin films were synthesized using AA‐CVD, where zinc acetate (Sigma Aldrich) and methanol (Fisher Scientific) were used as the precursor and solvent, respectively. N_2_ was also used as a carrier gas with a flow rate of 1 L min^−1^ in addition to the ultrasonic humidifier described above. The films were deposited using the same type of CVD reactor described above at a temperature of 450 °C on glass slides. Samples were not annealed after deposition.

*Synthesis of WO_3_ Thin Films*: Glass substrates (2.5 cm × 5 cm) were first cleaned with isopropanol and then with acetone, dried in air, and then placed inside the reactor, which was preheated to 300 °C for WO_3_ deposition. [W(CO)_6_] (0.06 g, 0.17 mmol) was dissolved in a mixed solution of acetone and methanol (15 mL, 2:1, Sigma‐Aldrich, ≥99.6%). The mixed tungsten solution was then transferred to a glass flask after 5 min in an ultrasonic bath. An ultrasonic atomizer/humidifier was used to generate aerosol droplets from the precursor solution and then further transported to the heated glass substrates via a flow of carrier gas nitrogen (300 cm^3^ min^−1^). The time taken to pass all aerosol droplets through the reactor was 30 min. The reactor was then allowed to cool to room temperature once the precursor solution was used up. The obtained substrates were annealed in an oven at 500 °C for 2 h in air, heated from room temperature at 5 °C min^−1^, and then allowed to cool to room temperature subsequently.

*Functionalized AuNPs*: 4‐mercaptobenzoic acid (MBA, Sigma Aldrich) was dissolved in ethanol (VWR Chemicals) to create a 1 × 10^−5^ m solution. 0.1 mL of the MBA solution was dropped into 0.5 mL of 40 nm citrate capped AuNP solution, purchased from SPI supplies. The solution was left overnight to allow MBA to functionalize the AuNPs by replacing the citrate capping layer. The solution was then centrifuged using an Eppendorf Minispin at 6k rpm for 6 min to cause the AuNPs to pellet. As much of the remaining solution as possible was removed leaving the NPs and deionized (DI) water was added to make a total volume of 1 mL, re‐dispersing the functionalized NPs. This process was repeated five times to remove any unbound MBA/citrate from the solution. After the final washing, enough DI water was added to have 0.5 mL of MBA‐functionalized AuNP solution.

*Substrate Preparation*: 1 µL of MBA‐functionalized AuNP solution was dropped onto the prepared films. The films were then placed on a hot plate which was heated with the substrate on it. Temperatures were limited to 100 °C, however samples were removed as soon as the droplet had evaporated, normally occurring after 15–30 s, before the hot plate had reached maximum temperature. A ring of AuNP of roughly 2 mm in diameter was visibly seen on the substrates.

To photo‐induce the PIERS effect for the decay studies, metal‐oxide films (TiO_2_, ZnO, and WO_3_) were pre‐irradiated under an UV lamp (UVItec LI 215G model (λ = 254 nm) UVC light) at a rough distance of 2 cm above the sample for around 1 h 45 min prior to AuNP deposition. This time was found to be long enough to induce a significant PIERS effect.

For the enhancement studies, samples were initially positioned to be focused under a Raman laser. After an initial SERS baseline spectrum was taken, samples were irradiated in position with UV light for 5 mins. The UV lamp was elevated around 4 cm from the samples such that UV rays were incident from the side and the top of of the sample. After the 5 minute activation time the UV lamp was briefly turned off for the time needed to conduct the Raman measurement and turned on again between measurements. The position of the sample and UV lamp were kept constant at all times.

*Characterization Techniques*: The Raman studies were carried out using a confocal Raman microscope (WiTEC) equipped with a He‐Ne laser (λ = 633 nm) with a bright field objective (Zeiss 100× NA 0.9) and an average power of 1 mW. The acquisition time of all Raman measurements was 5 s where three consecutive measurements were averaged for each spectra. The laser shutter was closed between measurements, limiting the exposure of the sample to the laser. Raman spectra were recorded immediately after irradiation and the addition of AuNP‐MBA, and then subsequently every 5 min until the enhancement was observed to return to the conventional SERS baseline. To determine average SERS spectra, ten measurements were taken across the sample at different positions.

X‐ray diffraction analysis was carried out using a Bruker‐Axs D8 (Lynxeye XE) diffractometer. The instrument was operated using a monochromated copper X‐ray source (Kα_1_, λ = 1.54 Å) under a glancing incident angle (θ) of 1°. Scanning electron micrographs were taken at 10 kV acceleration voltage, 7 mm working distance with a 30 µm aperture in a Raith eLine system.

## Conflict of Interest

The authors declare no conflict of interest.

## Supporting information

Supplementary
