# Supplementary material for: Dynamics of Photo‐Induced Surface Oxygen Vacancies in Metal‐Oxide Semiconductors Studied Under Ambient Conditions
Source: Adv Sci (Weinh). 2019 Sep 30;6(22):1901841. doi: 10.1002/advs.201901841 (PMC6864511; doi:10.1002/advs.201901841)
Supplement: Supplementary file 1 — Supplementary [file ADVS-6-1901841-s001.pdf]

Copyright WILEY-VCH Verlag GmbH & Co. KGaA, 69469 Weinheim, Germany, 2019.

Supporting Information

for *Adv. Sci.,* DOI: 10.1002/advs.201901841

Dynamics of Photo-Induced Surface Oxygen Vacancies in Metal-Oxide Semiconductors Studied Under Ambient Conditions

Daniel Glass, Emiliano Cortés, Sultan Ben-Jaber, Thomas Brick, William J. Peveler, Christopher S. Blackman, Christopher R. Howle, Raul Quesada-Cabrera, Ivan P. Parkin, and Stefan A. Maier**

Supporting Information

Dynamics of photo-induced surface oxygen vacancies in metal-oxide semiconductors under ambient conditions

Daniel Glass, Emiliano Cortés, Sultan Ben-Jaber, Thomas Brick, William J. Peveler, Christopher S. Blackman, Christopher R. Howle, Raul Quesada-Cabrera, Ivan P. Parkin, Stefan A. Maier**

Supplementary Figures

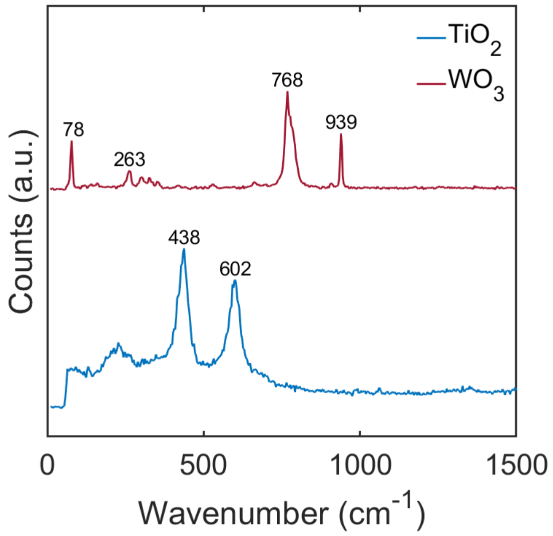

Figure S1. Raman spectra of blank TiO_2 (bottom) and WO_3 (top) show characteristic Raman bands for the respective metal oxide substrate. These bands were not always seen at low laser intensities when AuNP-MBA was deposited, assumed to be due to the intensity of the MBA bands dominating the spectra. No characteristic Raman bands were found the ZnO films.

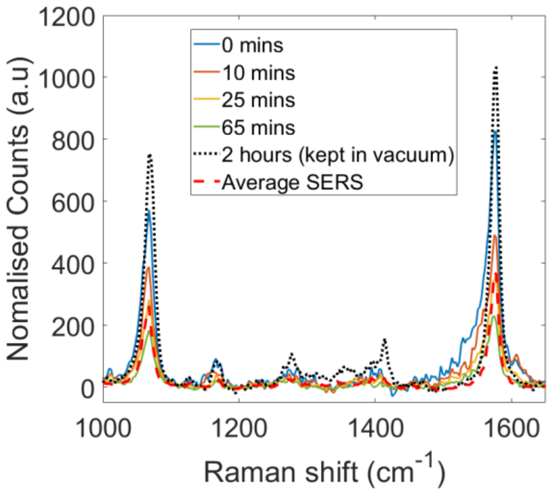

Figure S2. Raman spectra of MBA-AuNP on TiO_2 after UV irradiation (blue solid) and over time showing the enhancement changes from PIERS towards the average SERS (red dashed). Additional sample kept under vacuum after UV irradiation for 2 hours (black dotted) measured just after release from vacuum, shown to have comparable enhancements to the initial PIERS spectra as opposed to average SERS. Hence, exposure to air results in decay of PIERS enhancement.

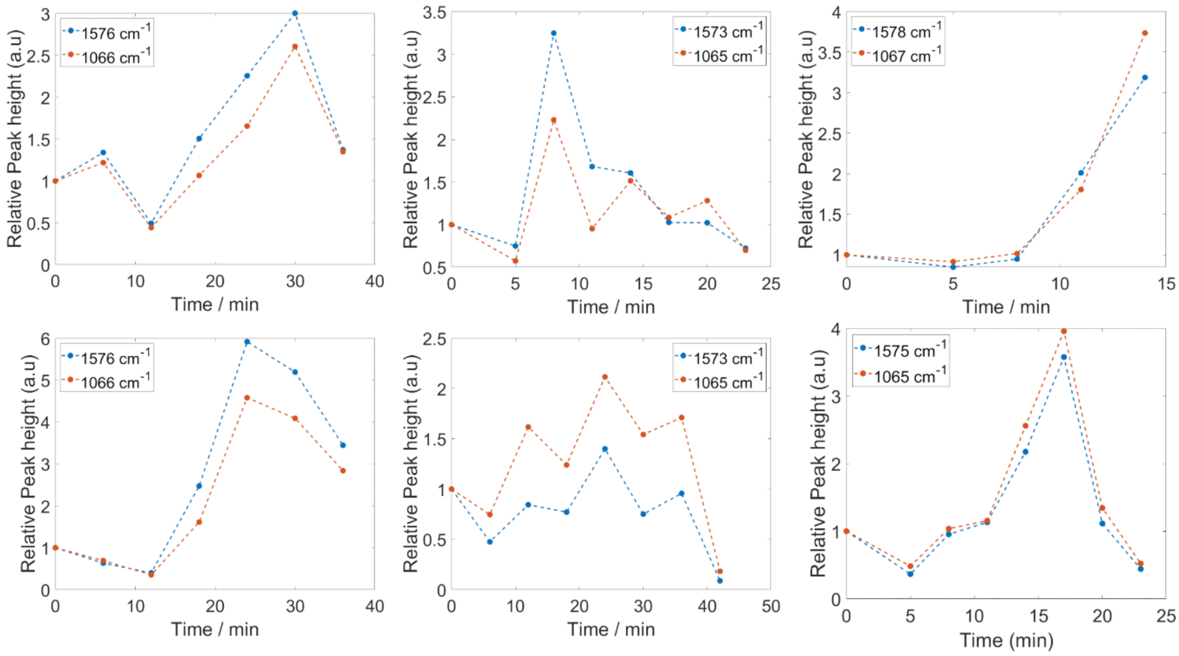

Figure S3. Examples of inconsistencies found in behaviour of MBA on ZnO samples under UV exposure. Relative intensity of MBA's main Raman bands over time, where each graph is a different position on different samples. A general trend of increase in intensity can

be seen for most samples, however noticeably fluctuations in intensity can be seen over short periods of time. Initial decrease in intensity between 0-5 minutes was assigned to laser induced degradation dominating over vacancy induced affects. Nearly all samples shown tend to decrease towards the end of the measured time (particularly > 15 minutes), which was attributed to subsequent healing of induced vacancies.

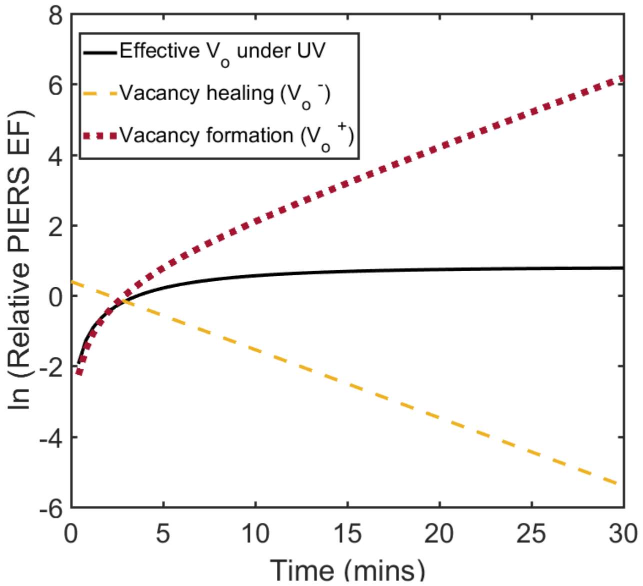

Figure S4. A sample of the calculated rate of vacancy formation using **Equation 2**, determined through deconvoluting experimental data. The rates shown here correspond to the measured data presented in **Figure 3**B and **Figure 3**C in the main text. Note, as described in the main text under no UV light exposure the change in effective vacancy concentration is only due to vacancy healing and therefore can be determined using **Equation 1**.

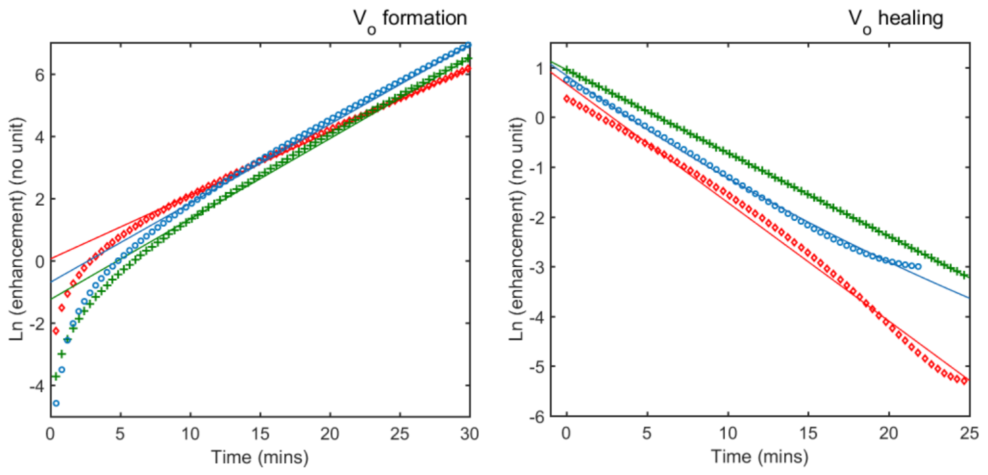

Figure S5. Logarithmic plots of enhancements due to oxygen vacancies over time with fits to linear region, used to calculate the rates of oxygen vacancy formation (left) and vacancy healing (right) in each respective metal oxide. Calculated rates appear in the main report, **Table 1**.

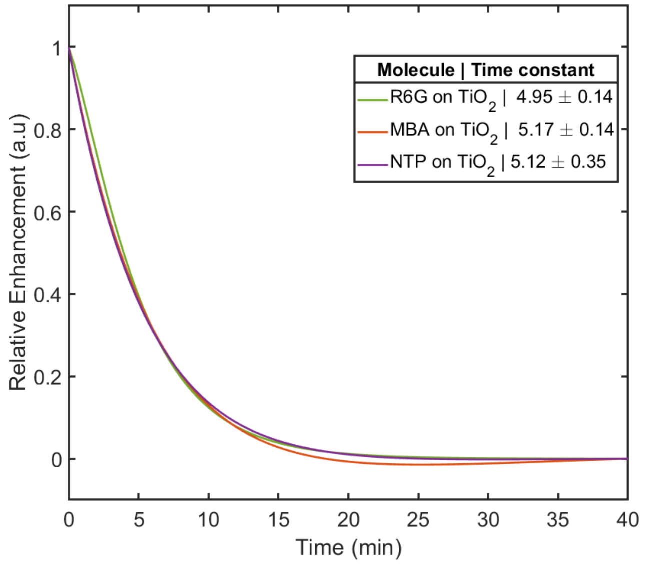

Figure S6. Calculated decay of PIERS induced probe molecule enhancement over time after UV exposure for MBA, 4-Mercaptobenzoic acid, R6G, Rhodamine 6G and NTP, 4-Nitrothiophenol on TiO_2 substrates. Relative decays were calculated from an average of PIERS decays over multiple positions on multiple substrates. Note, MBA was chemisorbed onto the AuNPS, whereas R6G and NTP were physisorbed onto the AuNP/TiO_2 substrate. No significant difference in the PIERS decay was noticed due to the type of molecule or adsorption.

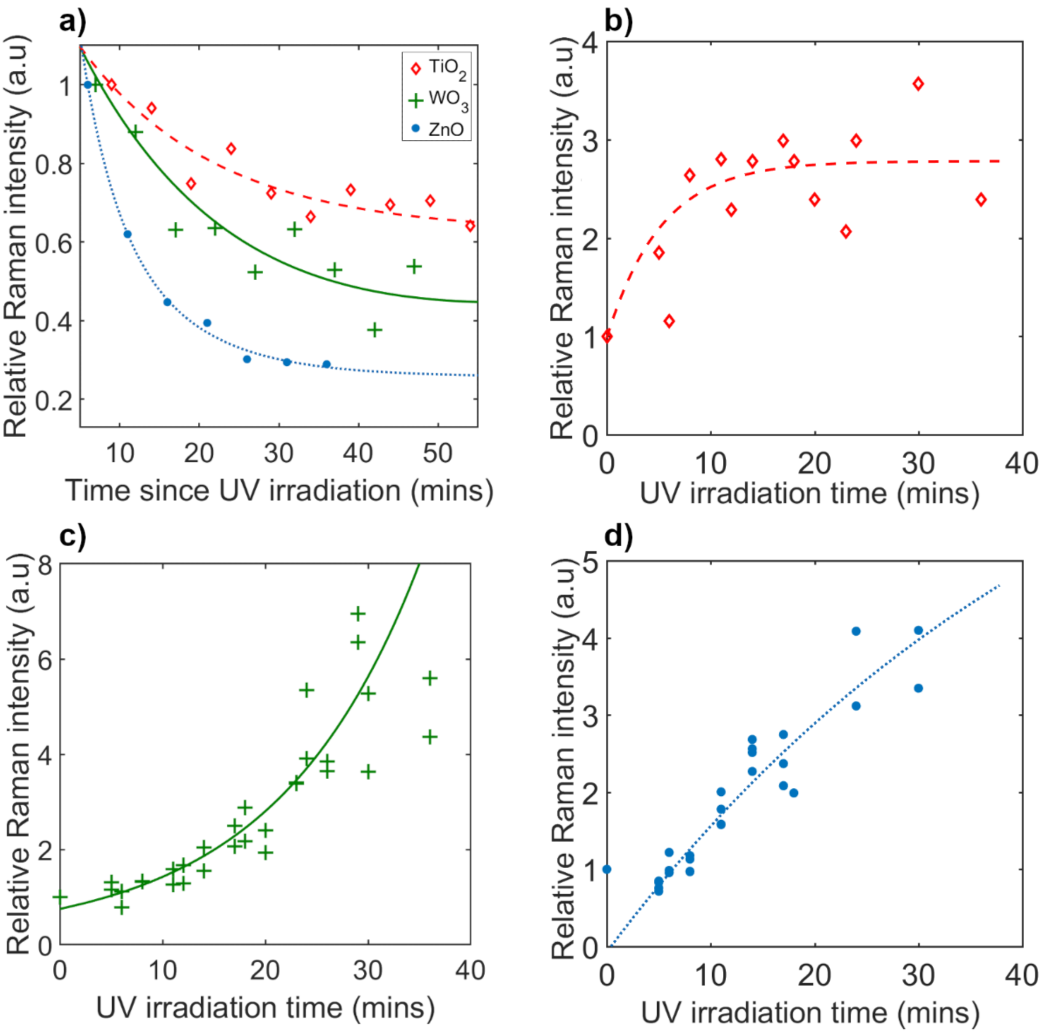

Figure S7. Average relative changes in main Raman band intensities of MBA upon different metal oxides, TiO_2 (red-dash, diamond), ZnO (blue-dot, dot) and WO_3 (green-solid, cross); after a UV irradiation period (a) and under constant UV exposure for TiO_2 (b), WO_3 (c) and ZnO films (d) respectively with respective fits.

Experimental Sections

Substrate Synthesis

Metal oxide (MO) films were grown using aerosol assisted chemical vapour deposition, see Methods, under specific conditions to produce flat thin films. Resultant films were annealed under ambient conditions at to create the desired crystalline phase and remove defects formed during film formation. Characterisation of the MOs was performed using X-ray diffraction (XRD), **Figure S8**, confirming that the films were produced in the desired

phase.

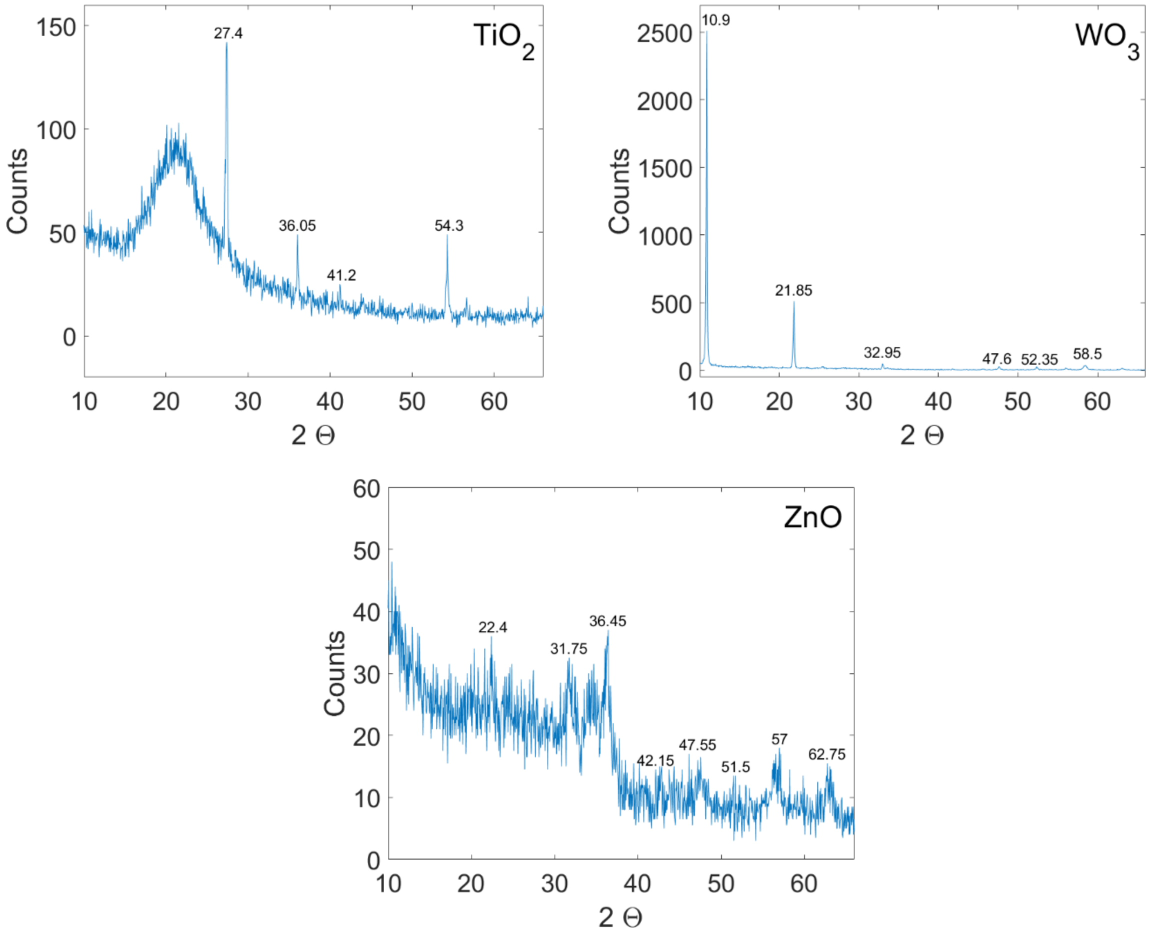

Figure S8. XRD patterns for TiO_2, WO_3 and ZnO. Notably ZnO films were the less crystalline.

Nanoparticle distributions

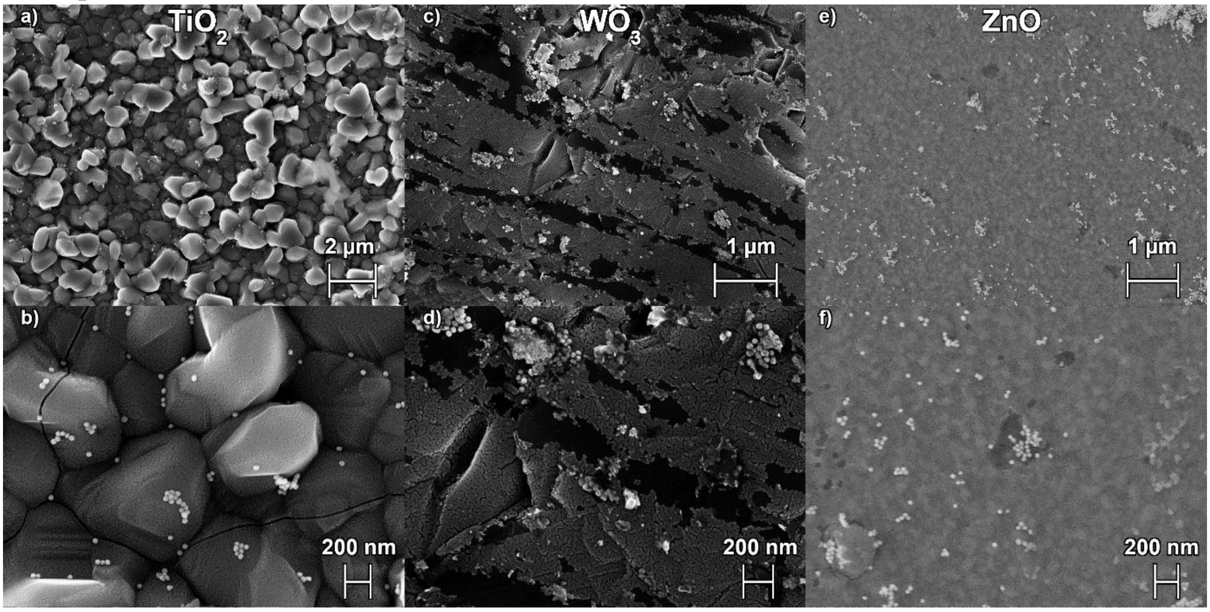

Figure S9. SEM images for TiO_2 (a,b), WO_3 (c,d) and ZnO (e,f) at low magnification (top row) and high magnification (bottom row) with AuNPs. Notably the images show ZnO films have a much smoother surface microstructure, which can have a significant effect on the photocatalytic activity of the films.

From SEM images NP were sized at 40 nm in diameter. AuNP appeared most distributed on TiO_2 with much less clustering. Large clusters were often found on ZnO films, as can be seen in **Figure S9**f. WO_3 films were found to cause AuNP to cluster less than ZnO but more than TiO_2 films.

Calculating oxygen vacancy lifetimes

All Raman spectra of MBA were found to have 2 significant Raman bands at a frequency shift of around 1060 cm^{-1} and 1575 cm^{-1}. A series of Raman spectra over time for AuNPs-MBA (AuNP functionalised with MBA) were taken on each metal oxide substrates after pre-irradiation and under constant UV irradiation. Reference spectra were taken before UV irradiation. Raman band intensities over time for these 2 bands were calculated using Matlab from the series of spectra, with the assistance of “findpeaks” function. The changes in band intensities were then normalized with respect to the SERS baseline. An average over the relative changes in enhancement were taken over all measured positions on each respective sample and then plotted against time.

The resultant plot was computationally fitted using Matlab's "Curve Fitting" toolbox. We refer the reader to the main manuscript where an explanation and justification for the type of functions used for analysis is explained in depth. Samples without UV irradiation were initially fitted to an exponential function in the form of

$$L(t) = a \times e^{(-b \times t)} + c \quad (1)$$

where t is time from initial measurement and a, b and c are fitting parameters. An average of each data for each MO was used to determine the average decay due to laser induced affects. This function describing laser induced signal decay was labelled L.

Band intensities were then determined using the same method above for pre-irradiated samples. The data was processed using the same method, however the functions were fit to

$$P(t) = V_o^{-}(t) + L(t) \quad (2)$$

where P is the measured PIERS data and V_o^- represents the changes in intensity due to oxygen vacancy healing. As described in the main text, **Equation S2** represents the case where, as no UV irradiation is present, no additional vacancies are induced, and therefore the change in effective induced vacancies is only due to vacancy healing. The contributions to the decay from laser photobleaching effects are independent to the photo-induced enhancements, hence the decay can be fit using a linear combination of these. V_o^- was then determined using calculated L values and was fit to an exponential function as described above with L. Parameter b is defined as the rate of each process and related to the time constant for the exponential decay. From this value, an estimation of the vacancy healing rate and lifetime was determined. An average for the calculated lifetime between each MBA peak, 1060 cm^{-1} and 1575 cm^{-1}, were taken for each MO. The reported data reflects an average of the lifetime estimations across all measurements for each substrate material.

Series of Raman spectra during UV exposure were initially fit to an exponential function of the same form as **Equation S1**. This was then analysed as described in the main text using **Equation 1** and **Equation 2** to calculate rates for V_o formation, V_o^+.

Extrapolated and shifts in induced Vo concentrations

It is important to note that the out of the three fitting parameters used above only ‘b’ has a physical interpretation – the rate. This is due to the normalisation process used to compare each respective process on each respective metal oxide. Hence, the resultant curves may be translated without affecting the rate of decay or increase respectively. Data was presented relative to the SERS baseline background. Hence, where no UV irradiation was used the decay in the measured SERS enhancement, due to laser photobleaching, was set so EF=1 when time=0. For samples pre-irradiated with UV, at long time periods where all the induced surface vacancies are healed the enhancement returns to the SERS baseline. However, photobleaching of the sample will also have occurred during measurements. Hence, at large t the enhancement would be comparable to the decrease in enhancement just due to laser photobleaching and so was shifted in y accordingly. For samples under UV irradiation, at time=0 no vacancies have been induced, and therefore the enhancement seen in purely due to the SERS baseline. Where the relative EF=1, the CE=0 (due to V_o). Hence, where CE and the effective vacancies are displayed in **Figure 3**D, at time=0 and later times after the UV lamp was turned off CE=0. V_o^- values were shifted in time to be 10 minutes after V_o^+ values. This corresponded to the average time samples were under ambient conditions after the initial long UV exposure before measurements were taken, required due to experimental constrains (i.e. deposition of AuNPs-MBA and aligning and focusing samples with the Raman laser). Data was also extrapolated between V_o^+ and V_o^- points. Extrapolated data was found using Matlab’s “pchip” function, a cubic spline data interpolation function.
